# Primary Tumor Location as a Prognostic and Predictive Marker in Metastatic Colorectal Cancer (mCRC)

**DOI:** 10.3389/fonc.2020.00964

**Published:** 2020-06-16

**Authors:** Ankur Bahl, Vineet Talwar, Bhawna Sirohi, Prashant Mehta, Devavrat Arya, Gunjan Shrivastava, Akhil Dahiya, K. Pavithran

**Affiliations:** ^1^Department of Medical Oncology, Max Super Speciality Hospital, New Delhi, India; ^2^Department of Medical Oncology, Rajiv Gandhi Cancer Institute and Research Centre, New Delhi, India; ^3^Department of Medical Oncology, Asian Institute of Medical Sciences, Faridabad, India; ^4^Medical Affairs, Dr. Reddy's Laboratories Limited, Hyderabad, India; ^5^Department of Medical Oncology, Amrita Institute of Medial Sciences and Research Centre, Kochi, India

**Keywords:** adenocarcinoma, Panitumumab, Cetuximab, Bevacizumab, colorectal cancer (CRC), tumor sidedness, primary tumor location, anti-EGFR mAb

## Abstract

Clinico-pathological differences between adenocarcinoma in the right and left colo-rectum play a role in determining the prognosis and response to treatment. Studies suggest that primary tumor location is more relevant as the disease progresses and reflects a possible difference in biology and response to therapy. This review aims to explore the clinico-pathological features of right and left colo-rectum and the impact of primary tumor location on prognosis of CRC as well as discuss the available clinical data on tumor sidedness in metastatic colorectal cancer. In so far as the clinical data of tumor sidedness is concerned, very few reviews have discussed the clinical implications of sidedness in heavily pre-treated metastatic colorectal cancer (second and subsequent lines of therapy in metastatic disease). This review aims to fill the current gap in this setting.

## Introduction

Colorectal cancer (CRC) is the third most common cancer globally, with an estimated 1.8 million new cases and 881,000 deaths occurring in 2018 (1). In India, CRC comprises 6.3% of the all cancers with annual incidence rates (AARs) of 4.4 per 100,000 for men and 3.9 per 100,000 for women (2). In terms of the left vs. right sided colorectal cancer, a 2017 study from Mumbai, India noted 80.2% cases originated from the left side whereas 19.8% from right side (3).

Embryologically, the right colon develops from the midgut while the left colon from the hindgut. These different origins consequently lead to differences in gene expression, methylation signatures, and the mutation profiles in right vs. left colorectal cancer (4). Right sided colonic tumors are more likely to have microsatellite instability, associated with a RAS or a BRAF signature, have a serrated pathway, and to have a JAK-STAT gene signature. Left sided colorectal tumors are more likely to be associated with WNT and MYC pathways activation, have beta-catenin activation, and are associated with EGFR and HER2 upregulation (5).

Multiple studies have highlighted the impact of sidedness on survival and suggested that primary tumor location (PTL) may be a predictive marker for treatment selection in metastatic colorectal cancer (mCRC) (6, 7).

This review will explore the differences in clinical and molecular characteristics between Right sided colon cancer (RCC) and Left sided colorectal cancer (LCC) along with the therapeutic and prognostic implications of various targeted therapies, especially anti-EGFR monoclonal antibodies, for the treatment of metastatic CRC (mCRC).

### Tumor Sidedness: Clinicopathological and Molecular Characteristics

Right sided Colonic cancers and Left sided colorectal cancers harbor different epidemiological, clinical, and molecular-pathological features (4, 8). Mucinous, undifferentiated, and signet ring histology is more common in right sided tumors as compared to left sided tumors (8). A 2018 SEER Database analysis of 163,232 colorectal patients revealed that 12.13% patients with Right sided tumors had a mucinous histology. In left sided tumors, 6.02% had a mucinous histology. Similarly the signet ring histology, albeit rare, was more common in the right sided cases as compared to left (1.43% right sided cases, 0.66% left sided cases) (9). This analysis also reported that Left sided tumors were more likely to be detected at a smaller tumor size than Right sided cases (median tumor size: 40 vs. 45 mm), *P* < 0.001 (9).

Due to relatively poor prognostic features, patients with Right sided tumor have poorer survival as compared to LCC (6–9). However, the impact of primary tumor location (PTL) on clinical outcome is more relevant following the development of metastatic disease, which reflects a possible difference in biology and response to therapy. The hypothesis is supported by multiple studies in metastatic settings, showing worsening in Overall survival (OS) in cancers originating from the right colon (6, 7, 10). Studies have not reported a significant difference in survival between left sided and right sided tumors in earlier stages (Stage I–III) (8). In fact, some studies have even reported a better survival for Stage I and II RCC (11).

#### Molecular Characteristics and the Impact of Sidedness

A study by Glebov et al., in 2003 used cDNA microarray technology and evaluated the difference between gene signatures of right and left sided colorectal cancer. This study reported more than 1,000 differentially expressed genes between right and left colon, with >2- and >3-fold differences in expression of 165 and 49 genes, respectively (5). A recent study by Loree et al. at MD Anderson Cancer Centre reported higher rates of BRAF, PIK3CA, CTNNB1, SMAD, KRAS, NRAS mutations, CpG island methylator phenotype (CIMP) and Mismatch repair defects in right sided tumors whereas TP53 mutations were more common on the left sided colonic and rectal tumors (12). In another study by Salem et al. 10,570 colorectal tumors were profiled using next-generation sequencing, immunohistochemistry, and other similar techniques (13). Sidedness could be determined for 2,413 tumors. BRAF mutations were reported in 25% right sided tumors whereas only 7% left sided tumors had BRAF mutations. Among other mutations, TP53 and APC were more commonly found on the left side whereas PIK3CA, CTNNB1, ATM, PTEN, and BRCA1 were more commonly mutated on the right side. Similarly, Mismatch repair (MMR) defects were more commonly seen in the right side tumors (22.3% of all right sided tumors) as compared to left (7.1%). Her2/neu gene amplification was reported in 5.4% rectal tumors and overexpression was reported in 2.7% rectal tumors. The amplification and ovexpression was relatively lower in both right (1.3% and 1.4% respectively) and left sided colonic tumors (2.8% and 1.7% respectively) (13).

The Consensus Molecular subtypes (CMS), initially proposed by Guinney et al. (14) in 2015, also show a differential expression based on tumor location, the CMS1 being more common the right side and CMS2 predominates on the right side (12). In a study by Loree et al., CMS1 subtype was reported in 36% right sided colonic tumors, whereas only 3–4% of left colorectal cancers had this subtype. CMS2 subtype was found in 56% left colonic, 61% rectal, and only 29% right colonic samples (12). An exploratory analysis of FIRE-3 study also reported similar results, where CMS1 subtype was more common on the right sided and CMS2 subtype was more common on the left. There was marginal difference in the distributions of CMS3 and CMS4 subtypes (15).

The differential distribution of CMS subtypes may offer greater insights into the drivers and pathophysiology of right and left sided colorectal tumors. Guinney et al. observed that CMS1 phenotypes were generally hypermutated and had lower somatic copy number alterations and relatively widespread hypermthylation signatures (12). MSI high tumors (common on the right side) are driven by CMS1 subtype and display strong activation of immune evasion pathways. In this analysis by Guinney et al., BRAF mutations more frequently occurred in CMS1 subtype and on the right side. CMS2 phenotypes reported higher somatic copy number alterations and consequently higher chromosomal instability along with upregulation of WNT and MYC downstream pathways. CMS3 phenotypes displayed low frequency of somatic copy number alterations, about 30% were hypermutated and a higher frequency of CpG Island Methylator Phenotype (CIMP). CMS3 phenotype was characterized by increased expression of various metabolic signatures which was reported in line with the presence of activating mutation in RAS (12). Guinney et al. also reported the prognostic relevance of CMS subtypes with regards to tumor sidedness. The study reported that CMS1 subtype was more common on the right side and CMS2 subtype was more common on the left. CMS2 subtype had a better survival after relapse whereas the prognosis was poorer in CMS1 phenotype after relapse (12).

### Tumor Sidedness: Implications for Upfront First Line mCRC Management

Biologics, in combination with chemotherapy, are indicated for the treatment of Unresectable metastatic Colorectal cancer (16). Even though there is clinical data for Biologics in borderline resectable and liver limited metastatic CRC, this review would not discuss this data (unless and until it is relevant to the discussion of tumor sidedness). Biologics indicated in upfront metastatic colorectal cancer setting include vascular endothelial growth factor (VEGF) inhibitor, Bevacizumab, and anti-epidermal growth factor receptor inhibitors, Cetuximab and Panitumumab (16).

Cetuximab is a chimeric monoclonal antibody whereas Panitumumab is a fully humanized monoclonal antibody.

## Is Sidedness Predictive for Anti-EGFR Monoclonal Antibodies in First Line Treatment?

The CALGB/SWOG 80405 trial was one of the pioneer trials to evaluate the difference of primary tumor location (PTL) and response to anti-EGFR monoclonal antibodies in patients with KRAS wild-type mCRC (17). In this trial, patients with KRAS wild-type (codons 12 and 13) mCRC received FOLFIRI or mFOLFOX6 and were randomized to either Cetuximab or Bevacizumab. Primary tumor location based retrospective analysis of this trial showed significantly prolonged median overall survival (OS) in patients with left sided tumors as compared to right sided tumors, irrespective of allocation to Cetuximab or Bevacizumab group. The median OS with Cetuxumab based therapy was 37.5 months in left sided tumors as compared to 32.1 months with Bevacizumab based therapy on the left sided (HR = 0.77, *p* < 0.04) (17). On the right side, Bevacizumab arm reported an OS of 24.5 months vs. an OS of 16.4 months in the Cetuximab arm. This retrospective analysis concluded that primary tumor location could be an independent prognostic factor in addition to predicting response to Cetuximab therapy (17).

Retrospective analyses of the FIRE-3 and CRYSTAL Phase III studies in RAS wild type (KRAS and NRAS) population confirmed the sidedness findings of CALGB 80405 (18). Cetuximab plus FOLFIRI were compared with FOLFIRI alone in the CRYSTAL trial whereas Cetuximab plus FOLFIRI were compared to Bevacizumab plus FOFIRI in the FIRE-3 study. In left sided tumors in Phase III CRYSTAL study, Ceuximab plus FOFIRI reported an OS of 28.7 vs. 21.7 months with FOFIRI alone (HR = 0.65, *p* = 0.002); whereas on the right side the OS difference was not statistically significant (18).

Retrospective analysis of FIRE-3 study revealed a statistically significant OS advantage for Cetuximab plus FOLFIRI arm in left sided tumors, OS of 38.3 months in Cetuximab arm vs. 28.0 months in Bevacizumab arm (HR = 0.63, *p* = 0.002). There was no difference in OS between arms when right sided tumors were analyzed, Cetuximab arm reported an OS of 18.3 vs. 23.0 months (HR = 1.31, *p* = 0.28) (18).

Similar results were also reported by the *post hoc* analysis of the pivotal trials of Panitumumab (PRIME and PEAK), showing improved PFS and OS in RAS wild type (KRAS and NRAS) left-sided tumors after the addition of Panitumumab to chemotherapy. PRIME study compared FOFOLX alone to the combination of FOLFOX plus Panitumumab whereas PEAK study compared the combination of Panitumumab plus FOLFOX vs. Bevacizumab plus FOLFOX (19).

In 2017, Arnold et al. (20) published the results of a retrospective pooled analysis from six randomized trials of tumor sidedness and anti-EGFR therapy in patients with RAS wild type (KRAS/NRAS wild type) metastatic colorectal cancer. Of six trials on anti-EGFR therapy, five trials were from first-line therapy (CRYSTAL, FIRE-3, PRIME, PEAK, and CALGB/SWOG 80405) and one trial from second-line therapy (Panitumumab's 20050181 trial). The results showed a significantly worse prognosis in patients with Right sided cancers, HR for OS in right-sided vs. left-sided tumors was 1.38 (95% CI = 1.17–1.63). In patients with left sided cancers, chemotherapy (either FOLFOX or FOLFIRI) plus anti-EGFR therapy (cetuximab or panitumumab) was associated with improved OS compared with chemotherapy with or without Bevacizumab (HR = 0.75; 95% CI = 0.67–0.84). However, no benefit of anti-EGFR therapy was seen in patients with right sided CRC (HR = 1.12; 95% CI = 0.87–1.45) (20).

Holch et al. (21) performed a meta-analysis of 13 first-line randomized controlled trials and one prospective pharmacogenetic study in metastatic mCRC. In this analysis; all first line anti-EGFR vs. anti-VEGF studies in RAS wild type mCRC patients revealed a significant OS benefit of anti-EGFR therapy in left sided tumors (HR = 0.71, *p* = 0.0003). An non-significant OS favoring anti-VEGF (HR = 1.3, *p* = 0.081) in patients with right sided tumors was observed (21).

The recently published pre-planned retrospective analysis of Panitumumab's VALENTINO trial also reported benefits in ORR, PFS, and OS with Panitumumab based induction therapy for left sided tumors (22). VALENTINO trial looked at the PFS noninferiority of maintenance with single-agent Panitumumab vs. Panitumumab plus FU plus Leucovorin after an induction treatment with Panitumumab plus FOLFOX in patients with RAS wild-type mCRC. Interestingly, the PFS benefit with Panitumumab plus FU plus Leucovorin during maintenance treatment was independent of tumor sidedness (22).

## Is Sidedness Predictive for Bevacizumab in First Line?

Whereas there is evidence to the effect that tumor sidedness may predict response to anti-EGFR monoclonal antibodies (17–22), the evidence specifically for Bevacizumab is not very robust. In all the anti-EGFR vs. anti-VEGF studies referred to in the above discussion (17–19), there was no statistically significant survival advantage of Bevacizumab over anti-EGFR antibodies in right sided tumors.

In a retrospective analysis of TRIBE trial by Cremolini and colleagues, which looked at intensification of frontline therapy in mCRC with FOLFOXIRI plus Bevacizumab, right sided mCRC had inferior OS as compared to left side (23.7 vs. 31.0 months, HR = 1.42, *p* = 0.010) (23). However, when the associations were adjusted for BRAF and RAS status, the right and left sides did not differ in terms of overall survival. Right sided patients benefitted more from the intensification of their frontline treatment, with both PFS and OS advantage (23).

A meta-analysis by You et al. (24) investigated the impact of tumor sidedness on Bevacizumab based treatment. This study reported a PFS benefit for patients taking Bevacizumab based treatment in left sided mCRC as compared to right (HR = 0.31, *p* = 0.03).

### Tumor Sidedness: Implications on Second and Subsequent Lines of mCRC Management

A retrospective analysis by Boeckx evaluated the effect of primary tumor location on second- or later-line treatment outcomes in patients with RAS wild-type mCRC, Study 20050181, and Study 20020408 were included in this analysis (10).

In study 20050181, the addition of Panitumumab to FOLFIRI did not result in an improved PFS or OS on the left sided RAS wild type tumors. For left side, Median OS was 20.1 in the Panitumumab arm vs. 16.6 months in the FOLFIRI arm (HR = 0.96; *p* = 0.7388) and PFS was 8.0 vs. 5.8 months (HR = 0.88; *p* = 0.3086). In right-sided mCRC patients, there were no significant difference between the groups (10).

In study 20020408, there was a significant PFS benefit in the Panitumumab plus BSC arm (5.5 vs. 1.6 months; HR = 0.31; *p* < 0.0001) in left sided tumors. However, in the right side there was no difference in PFS between arms (1.7 vs. 1.5 months; HR = 0.50, *p* = 0.1029) (10).

A retrospective analysis of phase III NCIC CO 17 trial compared Cetuximab with best supportive care (BSC) in patients with KRAS wild-type, chemotherapy-refractory disease was carried by Brule et al. (25). In this trial, Cetuximab significantly improved OS in left sided KRAS wild type tumors (median OS 5.4 vs. 1.8 months, HR = 0.28 [0.18–0.45], *p* < 0.0001). There was no difference in OS between the two arms in right sided KRAS wild type tumors (25).

A Korean single center study retrospectively investigated the impact of tumor sidedness in chemo-refractory mCRC patients on treatment with Regorafenib (26). There was significant benefit in PFS with Regorafenib in all left sided tumors (PFS 2.6 vs. 1.9 months, *p* = 0.04, respectively). In the subpopulation of KRAS wild type patients, PFS benefit was again significant with Regorafenib in left sided tumors (2.9 vs. 2.1 months; *p* = 0.04) (26).

## Guidance on Sidedness in Second and Subsequent Lines in Metastatic Colorectal Cancer (mCRC)

The NCCN Guidelines Panel has laid down a consensus statement with respect to selection of anti-EGFR antibodies and tumor sidedness in the treatment of first line metastatic CRC (16). The Panel recommends anti-EGFR monoclonal antibodies only in the setting of left sided RAS wild type tumors. However, the Panel is largely silent on the conditions or circumstances where anti-EGFR monoclonal antibodies could be used in right sided first line mCRC. ESMO CRC Guidelines advocate Early tumor shrinkage (at 8 weeks) as a reasonable exception which could warrant usage of anti-EGFR agents in right sided first line mCRC treatment (27).

Contrary to recommendations in first line mCRC, there are no recommendations on the relevance of tumor sidedness in second and subsequent lines of treatment in mCRC. The NCCN panel does make a passing statement that there is not enough evidence to use tumor sidedness for treatment selection in these settings. However, a well-defined guidance from either the NCCN Panel or the ESMO guidelines committee is warranted. Currently, if a patient receives Bevacizumab based treatment in first line RAS wild type mCRC (irrespective of sidedness), the next logical treatment and the relevance of tumor sidedness are both unanswered questions. The authors believe that a guidance in this domain may help in appropriate selection and sequencing of agents even in second and subsequent lines.

The retrospective analysis of a large second line Phase III 2000181 Panitumumab trial does not support the relevance of tumor sidedness. And at this point in time, evidence is not robust enough to consider tumor sidedness as a predictive marker in second and subsequent lines of mCRC.

## Criticism of Tumor Sidedness' Clinical Data

Despite the presence of extensive data on tumor sidedness in mCRC, it is undeniable that all the clinical data has emanated from retrospective analyses of Phase II/III clinical studies. The need for prospective clinical data on tumor sidedness is a moot point.

Whereas the authors acknowledge that the existing tumor sidedness data is convincing, the planning, and conduct of clinical trials with inclusion of prospective tumor sidedness will only boost the validity of historical data.

## Conclusion

Retrospective analysis of multiple randomized phase II/III clinical trials points to the effectiveness of anti-EGFR therapy in Left sided RAS wild-type mCRC whereas the same have also reported a lack of benefit in right sided tumors. There is irrefutable clinical evidence that primary tumor location serves as independent prognostic as well as predictive biomarker of response to anti-EGFR monoclonal antibodies in first line mCRC treatment. However, there is no consensus with respect to the implications of tumor sidedness in second and subsequent lines of treatment. The authors discussed the existing clinical evidence in this setting and believe the concept of tumor sidedness may not hold true in this setting. There is certainly a need for a consensus statement in this space.

## Author Contributions

AB, KP, VT, and AD conceived and collected data for the manuscript, participated in drafting and revising the article and gave final approval. BS, PM, DA, and GS participated in drafting and revising the article and gave final approval.

## Conflict of Interest

AD is currently employed by Dr. Reddys' Laboratories Ltd., Hyderabad, India as a Medical Advisor, Medical Affairs. The remaining authors declare that the research was conducted in the absence of any commercial or financial relationships that could be construed as a potential conflict of interest.
